# Migration and differentiation of muscle stem cells are coupled by RhoA signalling during regeneration

**DOI:** 10.1098/rsob.230037

**Published:** 2023-09-20

**Authors:** Mirco Brondolin, Dylan Herzog, Sami Sultan, Fiona Warburton, Alessandra Vigilante, Robert D. Knight

**Affiliations:** ^1^ Centre for Craniofacial and Regenerative Biology, King's College London, Guy's Hospital, London, London SE1 9RT, UK; ^2^ Oral Clinical Research Unit, King's College London, London, London SE1 9RT, UK; ^3^ Centre for Stem Cells and Regenerative Medicine, London, London SE1 9RT, UK

**Keywords:** satellite cell, myogenesis, zebrafish, cell motility, mechanotransduction

## Abstract

Skeletal muscle is highly regenerative and is mediated by a population of migratory adult muscle stem cells (muSCs). Effective muscle regeneration requires a spatio-temporally regulated response of the muSC population to generate sufficient muscle progenitor cells that then differentiate at the appropriate time. The relationship between muSC migration and cell fate is poorly understood and it is not clear how forces experienced by migrating cells affect cell behaviour. We have used zebrafish to understand the relationship between muSC cell adhesion, behaviour and fate *in vivo*. Imaging of pax7-expressing muSCs as they respond to focal injuries in trunk muscle reveals that they migrate by protrusive-based means. By carefully characterizing their behaviour in response to injury we find that they employ an adhesion-dependent mode of migration that is regulated by the RhoA kinase ROCK. Impaired ROCK activity results in reduced expression of cell cycle genes and increased differentiation in regenerating muscle. This correlates with changes to focal adhesion dynamics and migration, revealing that ROCK inhibition alters the interaction of muSCs to their local environment. We propose that muSC migration and differentiation are coupled processes that respond to changes in force from the environment mediated by RhoA signalling.

## Graphical abstract

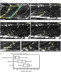

## 1. Introduction

Muscle is a highly regenerative tissue that relies on activation and mobilization of a resident stem cell population. In adult animals these are called satellite cells because of their peripheral location to muscle fibres where they lie beneath the myofibre basal lamina [1,2]. These muscle stem cells (muSCs) enable the highly regenerative capacity of muscle and act constantly throughout life to repair and replace damaged myofibres [3–6]. Impaired muSC function results in ineffective muscle repair, fibrosis and adipogenesis, associated with ageing, a number of muscle syndromes and inflammatory diseases [7]. Much of our understanding about the molecular regulation of muSC function comes from *in vitro* analyses in which cells are cultured in two dimension. It has become increasingly clear however, that cells display very different behaviours when cultured in two dimension compared to three dimension culture systems, with cells often showing activation and active migration. muSCs cultured in three dimension systems are also exposed to a different environment to that *in vivo,* lacking the complex extracellular environment and circulating factors that are critical for their regulation [8].

In all vertebrates quiescent muSCs express the paired domain transcription factor Pax7. After activation they continue to express Pax7 and the myogenic regulatory factor Myf5 concomitant with proliferation. Subsequently, muSCs express the pro-differentiation factors Myod and Myogenin and become committed to differentiation. Committed myoblasts align and fuse with each other to generate myotubes, or fuse existing myofibres [9,10]. The dynamics of this process are most clearly understood from freshly isolated mouse myofibres which, when plated *in vitro*, display activation and emigration of activated muSCs from beneath the basal lamina [11,12]. Proliferating muSCs *in vitro* display a rounded morphology, assume an elongated shape as they start differentiating and finally fuse to form multinuclear myofibres.

The function of Pax7-expressing myogenic progenitor cells in muscle regeneration appear to be conserved throughout the vertebrates and potentially within a number of invertebrate phyla [13]. In zebrafish a population of Pax7+ myogenic cells arise during development, generating the external cell layer at the periphery of the myotome [14,15]. Two populations of Pax7+ cells are present in the zebrafish myotome expressing *pax7a* and *pax7b* with some overlap [16,17]. Injury of adult zebrafish results in proliferation of Pax7+ muSCs and regeneration of small myofibres, as described in mammals [18,19]. Regeneration occurs through a stem cell-dependent approach in larval and adult stages and does not involve a de-differentiation process as described for axolotl [20,21].

*In vitro* analyses of muSC migration on myofibres revealed that cells show a rapid migratory behaviour [22,23]. Cell migration is classically described as amoeboid or mesenchymal [24]. Mesenchymal migration involves cell adhesion to the extracellular matrix via focal adhesions, with movement driven by actin driven protrusions at the leading edge and posterior retraction driven by cell cortex contraction [25]. Amoeboid migration is driven by a rapid cortical contraction and extension of the cell membrane which can occur in an absence of adhesions. Recent characterizations of cells cultured in three dimension matrices reveal a more complex picture of cell migration, with the classical descriptions representing extremes [26]. In particular, a lobopodial mode of migration was described for fibroblasts in which movement of the stiffer nucleus acts as a piston to drive forward motion [27]. High resolution imaging has suggested muSCs employ an amoeboid mode of migration with membrane blebbing driving a rapid movement along the myofibre [23,28]. This differs from descriptions of muSC migration *in vivo*, which suggests cells move in a mesenchymal manner by extending protrusions and utilizing adhesions. Multiphoton imaging of muSCs expressing GFP in Pax7:Cre-ERT2 mice reveal that muSCs show a polarized behaviour with extension of cell processes at the leading edge and retraction of the trailing edge in response to injury [29]. In an absence of myofibres following cell death, muSCs display a similar mode of movement, utilizing the surviving extracellular matrix surrounding the absent (or ghost) myofibre. This implies that interactions with the extracellular matrix provide traction for migration of muSCs. Descriptions of muSCs from *ex vivo* cultures of mouse limb muscle also reveal muSCs extending processes and extending by trailing edge retraction, indicative of a mesenchymal migratory behaviour [30].

Adhesions act to tether cells to the extracellular matrix, but also enable transmission of force to and from cells via the actinomyosin cytoskeleton. Several signalling proteins respond to force transduced via the cytoskeleton in myogenic cells, including Hippo signalling proteins Yap and Taz, the SRF activator MRTFA (MAL1/MKL1) and the MAPK activated ERK and p38 kinases [31–33]. Both Yap and Taz promote proliferation of activated muSCs [31,34,35].

In quiescent muSCs, force induced activation of RhoA via Wnt4 signalling acts to repress Yap activity so preventing muSC activation [36]. Migration of muSCs has been associated with a Wnt7a-driven activation of Rac [37] reflecting the oft-observed opposing functions of RhoA and Rac in regulation of cell behaviour. Descriptions of muSCs responding to needle stick injury in mice suggests that the transition from quiescence to activation by muSCs involves a switch from Rac to Rho signalling [32]. Differing descriptions for how RhoA and Rac signalling direct muSC homeostasis in the niche and their responses to injury may reflect different mechanical environments that locally induce mechanical signalling through the cytoskeleton. The response of muSCs to injury is dependent on their environment, specifically the force conveyed by substrate stiffness to cell adhesions linked to actinomyosin. Increased tension in muSCs as consequence of high substrate stiffness promotes muSC proliferation *in vitro*, particularly when the stiffness corresponds to that found in injured muscle [38,39]. This proliferative response to increased substrate stiffness corresponds with increased migration in a Yap dependent manner [40]. Notch signalling is a major regulator of muSC proliferation [41]. Notch signalling is regulated by stretch-induced Yap activity during fetal myogenesis to maintain an appropriate level of Pax7+ progenitor cells [42], revealing a mechanism by which mechanical signals can control of muSC activity. These observations suggest that mechanical induction of Yap activity mediated by the actinomyosin cytoskeleton will dictate whether an activated muSC will proliferate through regulation of Notch. It is therefore important to understand how forces experienced by muSCs *in vivo* are transmitted from the environment and the consequences for proliferation or differentiation.

Imaging of pax7a:egfp transgenic larvae has shown that following injury to the trunk myotome, GFP-expressing muSCs extend protrusions and migrate from the myoseptum into the myotome [16]. Characterization of muSCs in zebrafish has shown that these cells are resident at the myoseptum, which is enriched in molecules associated with extracellular matrix such as Fibronectin and Laminin [16,17,21,43,44]. The similar molecular composition of the zebrafish myoseptum and satellite cell niche in mammals suggests muSCs resident in the myoseptum are a progenitor population that responds to injury.

Following their activation muSCs undergo exit from the myoseptum and migrate to damaged myofibres in the myotome. Migration of muSCs to injuries in zebrafish appears to involve a polarized cell behaviour with protrusions, similar to behaviour of muSCs *in vivo* in mice [45]. How this migration to injured myofibres is regulated is unclear, but several assumptions can be made based on studies of other mesenchymal cells such as fibroblasts or mesodermal progenitor cells in zebrafish [46,47]. These include a polarized distribution of focal adhesions relative to the direction of migration, a dependency on myosin polymerization to induce cortical contractility driving retraction of the trailing edge, and extension of cellular processes in the direction of cell migration.

In order to determine how muSC migration is regulated *in vivo* we have asked how muSCs respond to inhibition of RhoA signalling using the well characterized ROCK inhibitor Y-27632. We find that cell shape and adhesion dynamics of muSCs are altered in response to ROCK inhibition. These are correlated with changes to cell movement that can be explained by a reduced mechanical interaction with the extracellular matrix. Changes to cell behaviour are correlated with diminished expression of cell cycle genes, reduced expression of Yap target genes and increased expression of myogenic differentiation inducing genes. Based on our observations we propose a model in which RhoA activity is required for cell migration through regulating adhesion formation and controlling cortical myosin contractility. Perturbation of ROCK-dependent cell migration leads to diminished proliferation and increased differentiation, suggesting that the interactions of migratory cells with their environment dictates cell fate during muscle regeneration.

## 2. Methods and materials

### 2.1. Animals and experimental procedures

All animals used were reared at the King's College London Zebrafish Facility. Adult wild-type AB or Tg(pax7a:GFP) [48] zebrafish (*Danio rerio*) were raised in Tecniplast Multi-Linking WTU systems, on a 14 h light/10 h dark cycle, in standard conditions [49]. Embryos were obtained by natural spawning and embryonic fish were maintained in E3 medium at 28.5°C. After 5 days post fertilization (dpf), larvae were fed with Gemma75 (Skretting) daily.

### 2.2. Injury experiments

To induce muscle injury and regeneration a sharpened tungsten wire was applied to the myotome of anaesthetized larvae embedded in low melting point agarose as previously described [50]. Either a single punctate injury to ventral myotome 13, or multiple injuries to myotomes 8–14, were performed depending on whether animals were used for imaging or for extraction of RNA.

Animals were exposed to 50 µM Y-27632 dihydrochloride (Tocris) 6 h prior to commencement of the experiment and throughout the experiment with replacement of drug at 12 h intervals. A 10 mM stock dissolved in DMSO was diluted in E3 medium to 50 µM. Control samples were incubated with 0.005% DMSO.

### 2.3. Immunolabelling and BrdU incorporation

To detect GFP, phospho-Paxillin, Vinculin or Myogenin, larvae were anesthesized in 0.02% MS-222, fixed in 4% paraformaldehyde (PFA, Sigma) overnight at 4°C and subsequently stored in 100% methanol (Fisher) at −20°C. Samples were washed in 0.1% Tween20 in phosphate-buffered saline (PBT, Sigma) and permeabilized. Samples were blocked in 5% goat serum (Life Technologies) in PBT for at least 2 h, then incubated with primary antibody diluted in 5% goat serum/PBT over night at 4°C. Samples were washed for at least 4 h in 0.1% PBT the following day, then incubated with secondary antibodies diluted in 5% goat serum/PBT for 2 h at room temperature. After several washes in PBT, samples were taken through glycerol series and mounted in VectaShield with DAPI (Vector Laboratories).

To label dividing cells with BrdU, larvae were exposed to 10 mM BrdU diluted into E3 medium and processed as previously described [16].

Antibodies used were chicken polyclonal anti-GFP (1 : 500; Millipore), rabbit polyclonal anti-GFP (1 : 500; Life Technologies), rabbit polyclonal anti-phospho-Paxillin (Tyr118) (1 : 200; Thermo Scientific), mouse anti-Vinculin (1 : 200; Sigma), rabbit polyclonal anti-Myogenin (1 : 50; Santa Cruz Biotechnology), rat monoclonal anti-BrdU (1 : 250; Abcam). Secondary antibodies used were Alexa conjugated antibodies diluted 1 : 500 in goat serum and PBT.

### 2.4. RNA isolation and qRT-PCR

Total RNA was isolated from dissected injured trunk regions of larvae at 24 h post injury (hpi). Tissue was homogenized in TriReagent (Sigma) and total RNA isolated according to the manufacturer's protocol. RNA concentration was measured using a Nanodrop spectrophotometer (Peqlab Biotechnology GmbH, ThermoFisher Scientific) and 500 ng of total RNA was reverse transcribed into cDNA using random hexamer primers (Promega) and M-MLV reverse transcriptase (Promega).

The C1000 TM Thermal Cycler with CFX384 Optical Reaction Module (Bio-Rad) was used to perform quantitative real-time PCR experiments. Amplification after each PCR cycle was detected via qPCRBIO SyGreen Mix Lo-Rox (PCR BioSystems). All PCR reactions were done as technical triplicates in 384-well plates in a total volume of 5 µl. Data were analysed with CFX Manager 3.0 software (Bio-Rad). Expression is always shown relative to the untreated controls and relative to *elf1α* as internal expression control. Expression data were calculated according to the ΔΔ*C*_t_ method [51]. Primers for qRT-PCR assays were tested for efficiency before use and results were considered for corrected expression values. Primer dimers formation was excluded by melt curve analysis.

### 2.5. Imaging acquisition and analysis

Images of fixed samples were acquired using a Leica SP5 scanning point confocal microscope with a 20x (NA 0.75) or a 40x oil (NA 1.25) objective.

Live imaging of zebrafish was performed using a Zeiss 7MP dual beam multiphoton microscope with Vision II and MPX lasers (Coherent). Samples were embedded in 2% low melting point agarose and immersed in E3 medium containing MS222 (Sigma) at 0.01% or lower. Z-stacks of sample images were acquired with XY image dimensions 353.55 × 353.55 µm (526 × 526 pixels) and Z intervals set to 1 µm every 25 min using a 20× water dipping objective (W Plan-APOCHROMAT 20X/ 1.0 NA DIC-VIS-IR).

Analysis of images was performed using Image J/ Fiji [52,53] or Imaris 9.1 (Bitplane AG) in three dimensions.

Analysis of phospho-Paxillin (pPaxillin) distribution in GFP+ muSCs was determined using Imaris. GFP+ muSCs were segmented using the *Surfaces* function (0.35 µm surface detail, constant absolute intensity threshold). pPaxillin puncta distribution and dimensions were determined using the spot function (1.2 µm estimated XY diameter, constant quality filter type) and those that were less than 0.6 µm from the edge of a GFP+ surface were analysed. The *Spots to Spots* closest distance function was used for determining pPaxillin+ puncta clustering.

Cell tracking and shape analysis was performed using Imaris with additional manual correction of segmentation where adjacent cells were clustered. Prior to cell tracking and analysis, all image stacks were converted into .ims format using Imaris file converter 9.2.1. Cell tracking and analysis were carried out using the *Surfaces* function. A surface grain size of 0.5 µm was identified as suitable for the surface function to adapt to the shapes and structures of the cells. The diameter of the largest sphere was set to 2.5 µm, in order to register all cells.

Due to variations in the fluorescence intensity between samples, the threshold values were manually chosen for each individual sample to differentiate between visible cells and background noise. Following thresholding, the lineage algorithm was chosen to track the cells using the following parameters: max distance = 5.00 µm, max gap size = 2 µm and track duration above 2.5 s.

Following automated tracking, manual correction was carried out to separate individual cells that were clustered as one object, and to combine tracks of cells that were not recognized by initial segmentation.

The following parameters from cell tracking were then exported for statistical analysis: track length [µm], displacement_delta [µm], track speed mean [µm s^−1^], track sphericity mean, track volume mean [µm^3^], track area mean [µm^2^], track ellipticity oblate mean and track ellipticity prolate mean, centre of mass (X, Y, Z) and velocity angle (X, Y, Z).

### 2.6. Statistical analysis

Measures of cell numbers acquired from samples processed by immunolabelling were tested for a normal distribution using a Kolmogorov–Smirnov test. Significance was analysed using Student *t*-test, 2-way ANOVA with Bonferroni *post hoc* correction, or Kruskal–Wallis by rank tests, as appropriate. All *p*-values are indicated in the figures (*p < 0.05; **p < 0.01; ***p < 0.001).

Measures of cell shape and movement were evaluated for normal distribution by Shapiro–Wilks tests. Statistical tests for differences in cell shape and movement using injury and treatment as covariates were performed by 2-way ANOVA or *t*-tests in R. Average values were normalized by the invnorm function using qnorm and ties.method for ranking of data and plotting as a distribution. Multivariate analysis was performed using principal component analysis (PCA), linear correlation and pairwise or multiple comparisons in R.

Multivariate analysis was performed using principal component analysis (PCA) and principal components examined for contributions of each variable of cell shape and movement.

Multiple linear regression models with mixed effects were generated explaining how variables of cell shape and movement were affected by injury or ROCK inhibition in a time dependent manner using the Stata v15 package [54]. A three-level mixed linear regression analysis with random intercepts at both animal number and cell ID was conducted to look at how cell shape and movement were affected by injury or ROCK inhibition over time. A separate analysis was conducted for each cell shape and movement variable (dependent variable) with time, injury, treatment and their interactions as the independent variables. To aid interpretation the injury and treatment variables were combined to create a new variable called injtrt where condition 1 = (uninjured, control), condition 2 = (uninjured, Y-27632), condition 3 = (injured, control), condition 4 = (injured, Y-27632). Interaction terms for time and injury/ Y27632 treatment were tested for significance and included in models if *p* < 0.05.

## 3. Results

### 3.1. Characterization of muSC migration

In order to determine how muSC migration relates to classic models of cell migration we sought to evaluate several key traits used to classify cell movement in muSCs responding to injury. To observe muSCs *in vivo* we used the zebrafish pax7a:egfp transgenic line [48]. This line expresses GFP in muSCs that can easily be observed as they respond to injury by migrating and regenerating myofibres [16,21]. We aimed to measure the following traits in GFP+ muSCs responding to injury: cell shape (rounded versus elongated), the presence or absence of cell adhesions and their distribution, migratory behaviour and cell polarity.

**Figure 1.**
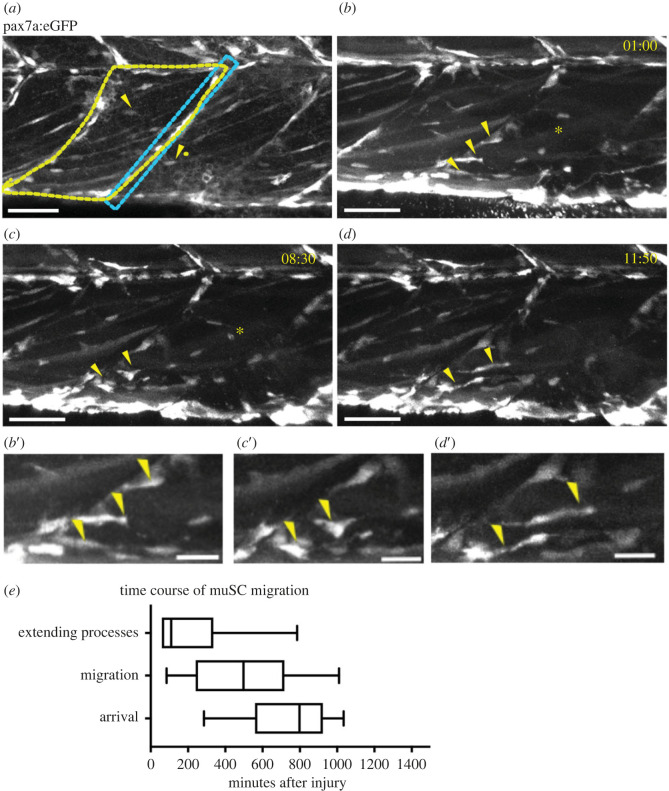
MuSCs show a transition in shape as they move from the myoseptum towards the site of injury (asterisk). GFP + muSCs (arrowheads) in uninjured 7 dpf pax7a:egfp larvae are located at the myoseptum (blue box) or in the myotome (yellow outline, *a*). In the presence of an injury GFP + muSCs at the vertical borders of the myoseptum extend processes into the myotome (*b*′, arrowheads) and undergo extensive movement along the myoseptum (*b*). They then stabilize a process extending into the myotome (*c*′, arrowheads) and detach from the myoseptum (*c*). Subsequently the GFP + muSCs migrate towards the site of injury, extending processes in multiple directions (*d*′, arrowheads) as they migrate (*d*). Quantification of cell responses to injury reveal three phases: extension of cell process, migration and arrival at the injury site (*e*, *n* = 28 cells from 6 animals). Scale bar 50 µm (*a–d*), 20 µm (*b*′–*d*′).

In uninjured 7 days post fertilization (dpf) larvae GFP+ muSCs reside at the borders or myoseptum of the myotomes in the trunk (figure 1*a*). Myofibres expressing GFP stretch across the myotome. Following injury, GFP+ cells at the vertical myoseptum extend processes into the myotome from 1 h post injury (hpi) and undergo extensive movement within the vertical myoseptum (figure 1*b,e*; electronic supplementary material, movie S1). Some cells will then stabilize protrusions into the myotome and detach from the myoseptum from 6 hpi (figure 1*c*,*e*). These cells show a change of shape from a flattened elongated appearance at the myotome boundary to a more rounded shape in the myotome. From around 6–8 hpi, cells in the myotome then extend processes and many of these will migrate towards the injury, with the majority of cell migration towards the injury completed by 15 hpi (*d*, *e*).

### 3.2. MuSCs display a polarized distribution of adhesion molecules

**Figure 2.**
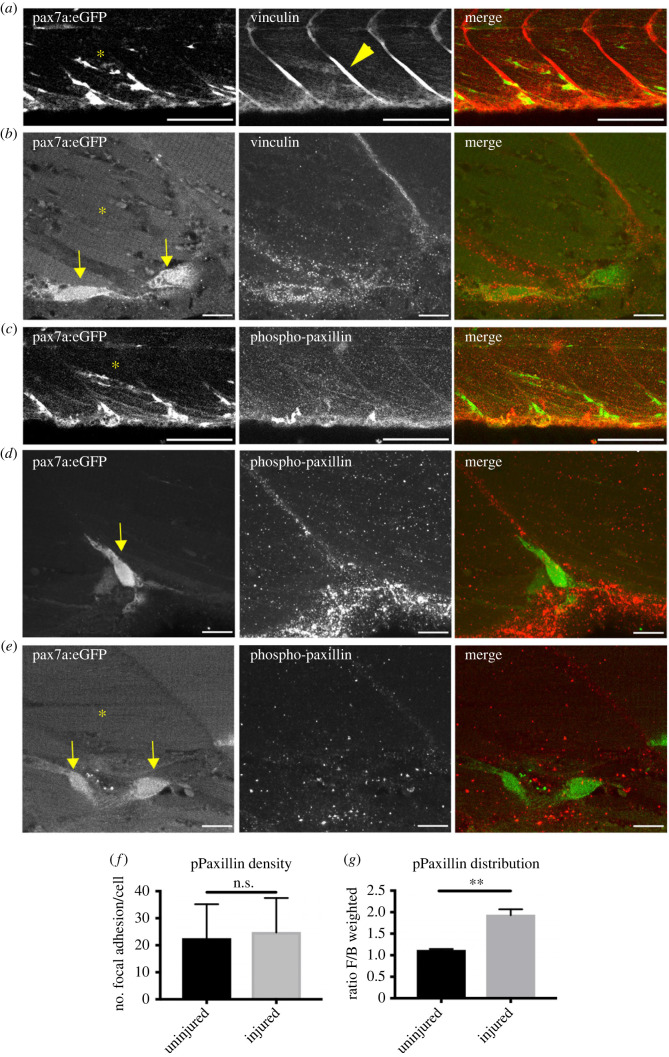
Adhesion molecule localization is polarized in muSCs migrating to injuries. Vinculin is detectable at the myoseptum (arrowheads, *a*) and is present as puncta on GFP+ cells (arrows, *b*) in injured (labelled by asterisk) pax7a:egfp larvae. The activated adhesion protein phosphoY118-Paxillin could be detected weakly at the myoseptum in pax7a:egfp larvae (*c*) and was present as puncta in GFP+ cells at the myoseptum (*d*) and within the myotome (*e*). Quantification of adhesion density within GFP+ cells was performed using Imaris with puncta defined as showing a diameter > 1.2 µm (*f*, *n* = 16 cells) and distribution of puncta was characterized by defining the ratio at the front compared to the back of a cell relative to the position of the nucleus (*g*, *n* = 16 cells). Statistical comparisons were performed by Student *t*-test and significance indicated (**p < 0.01, N.S. not significant) with *n* = 3 animals per condition. Scale bars 50 µm (*a,c*), 10 µm (*b,d,e*).

Cells displaying a mesenchymal behaviour use focal adhesions for generating traction as they migrate [55]. We therefore examined muSCs for the presence of focal adhesions and evaluated whether these were distributed in a polarized manner. The integrin binding protein vinculin is recruited to a variety of adhesions and is required for maturation of focal adhesions [56,57]. In uninjured animals at 7 dpf we observed high levels of vinculin at the myoseptum (figure 2*a*) as previously described [58,59]. There was little Vinculin labelling in GFP+ muSCs resident at the vertical myoseptum, but in muSCs resident within the myotome vinculin could be detected (figure 2*b*). In zebrafish there are two paxillin genes, paxillin-a and paxillin-b [60]. Both zebrafish paralogues show conservation of residue Tyrosine 118 with human PAXILLIN; when this residue is phosphorylated Paxillin is recruited to nascent focal adhesions, interacts with FAK and stabilizes the adhesion complex [61]. Phospho-Tyrosine118 (Y118) Paxillin (pPaxillin) labelling could be observed at the myoseptum although at lower levels than vinculin (figure 2*c*). Paxillin was also present throughout the myotome as discrete puncta, presumably within adhesions of non-GFP+ cells (figure 2*d,e*). In muSCs present within the myotome pPaxillin + puncta were present across the cell (figure 2*e*).

As pPaxillin is enriched at several types of adhesions, we classified antibody labelling according to the size of the labelled structure using Imaris. A threshold of 1.2 µm diameter for pPaxillin labelling is used to define focal adhesions in mammalian cells [62,63]. We applied this criterion to identify pPaxillin + puncta > 1.2 µm in diameter, then selected those that were present in GFP + muSCs by applying a cut-off for puncta less than 0.6 µm from the edge of a GFP+ cell. In uninjured larvae we observed an average of 25 puncta per cell and this was unchanged in larvae following injury (figure 2*f*).

As muSCs migrate to damaged fibres we predicted that they would be polarized and so show a differential distribution of pPaxillin relative to their axis of movement. By categorizing the distribution of puncta along their axis of orientation, relative to the position of the nucleus, we obtained a measure of polarized distribution. This revealed that the ratio of puncta present in the front compared to the back is higher in muSCs within injured myotomes compared to uninjured myotomes. This reveals that adhesions are distributed in a polarized manner in muSCs moving in response to injury (figure 2*g*, electronic supplementary material, figure S8).

### 3.3. ROCK inhibition perturbs muSC migration

RhoA activation of ROCK is necessary for both mesenchymal and amoeboid modes of cell migration. We evaluated the dependence of muSCs for ROCK activity during their response to injury by applying the small molecule inhibitor of ROCK, Y-27632 (electronic supplementary material, movies S1–S6). Cells were segmented and tracked using Imaris with manual correction where appropriate and measures of cell shape and movement determined (electronic supplementary material, movies S7–S12). To understand the consequences of inhibiting ROCK we evaluated when GFP+ cells at the myoseptum started to extend protrusions in response to injury, when they exit from the myoseptum and when they reached the injury. There was no significant difference between animals exposed to Y-27632 compared to control animals when comparing the time when cells started to extend protrusions towards the site of injury or when they became resident at the injury (electronic supplementary material, figure S1). However, muSCs appear to initiate migration earlier in animals treated with Y-27632 compared to injured control animals. This apparent discrepancy between an earlier onset of migration but no difference in arrival at the injury site between conditions could be explained by a delay in active migration of muSCs to the injury site, even though they are activated earlier, but this could not be determined from analysis of the time-lapsed movies obtained.

**Figure 3.**
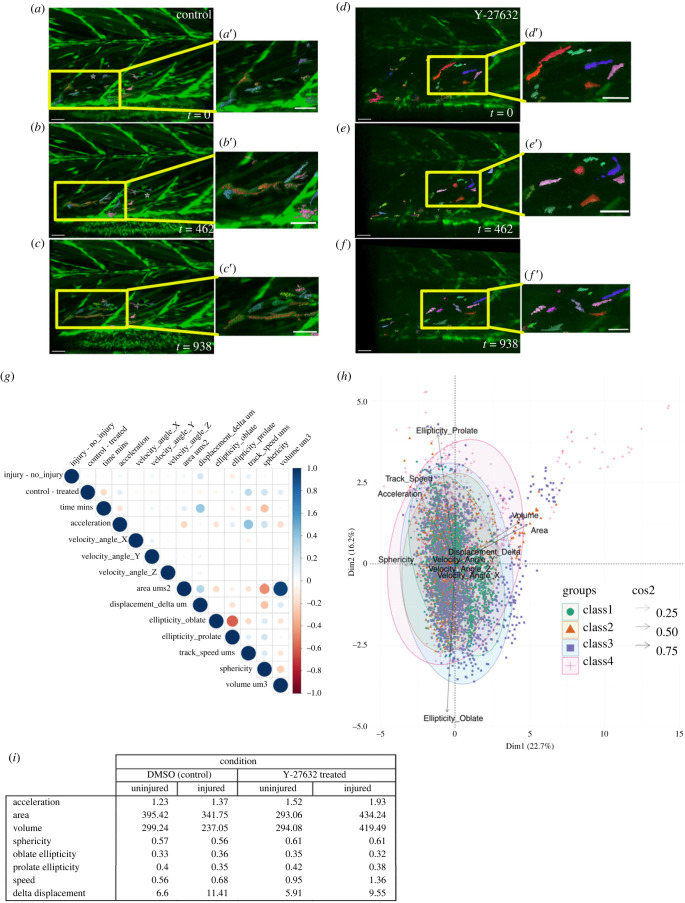
Inhibition of ROCK activity by Y-27632 results in changes to the shape and movement of muSCs responding to injury. Representative images from time-lapsed vidoes of control injured larvae (*a–c*) and experimental animals exposed to 50 µM Y-27632 (*d–f*) processed using Imaris to segment cells with inset images (yellow box) showing selected cells in the injured myotomes (*a*′–*f*′). Time-lapsed images were captured from 8 hpi and timestamp shown is minutes after start of the time-lapse. Each segmented cell is shown by different colour relative to the GFP signal (green). Measures of cell shape and movement were extracted by Imaris and compared by correlation analysis to identify positive (blue) or negative (red) correlations (*g*, *p* values represented by size) across all conditions (*g*). Principal Component Analysis was used to map relative contributions of variables of cell shape and movement to PC1 and PC2 (line density, cos) for the four classes of data (*h*, class 1: no injury, untreated, class 2: no injury, Y-27632, class 3: injury, untreated, class 4: injury, Y-27632). Average values of cell shape and speed are shown for each condition (*i*). Scale bars 30 µm (*a–f*, *a*′–*f*′).

In both untreated control and Y-27632 treated animals GFP+ cells responded to injury (figure 3*a–f*). We tested whether changes to variables of shape (volume, surface area, oblate ellipticity, prolate ellipticity, sphericity) or movement (delta displacement length, mean instantaneous speed, angle of velocity in *x*, *y* or *z* planes) were correlated with injury or inhibition of ROCK (figure 3*g*). Increased values of oblate ellipticity reflect cells assuming a more rounded, disc shaped morphology, whereas increased prolate ellipticity is indicative of cells assuming a more elongated morphology. Changes to area, volume and sphericity correlated with inhibition of ROCK, whereas changes to prolate ellipticity and to a lesser extent sphericity, were anti-correlated with injury (electronic supplementary material, table S1). Principal component analysis revealed PC1 and PC2 contribute approximately 39% of variance within the datasets (electronic supplementary material, figure S2). PC1 (22.7% variance) shows a high contribution for variables volume, area and sphericity, whereas PC2 (16.7% variance) has high contributions for variables ellipticity (oblate and prolate) and instantaneous speed (electronic supplementary material, figure S2).

To examine how injury and inhibition of ROCK interacted to affect cell shape and movement we defined four classes. These were uninjured (class 1), uninjured with Y-27632 added (class 2), injured (class 3), injured with Y-27632 added (class 4). Variables of cell shape and movement were plotted against PC1 and PC2 reflecting their relative contribution (figure 3*h*). When comparing these four classes across PC1 and PC2 it is apparent that inhibition of ROCK (class 2, 4) is associated with changes to cell volume, area and sphericity. By contrast, injury (class 3) is associated with changes to instantaneous speed and prolate ellipticity.

We investigated how injury and Y-27632 affected cell shape and movement using 2-way ANOVA (electronic supplementary material, table S2). This revealed that Y-27632 affected cell shape (area, volume, oblate and prolate ellipticity, sphericity) and movement (average speed, velocity-y). Injury affected some measures of cell shape (prolate ellipticity, sphericity) and movement (average speed). Interaction effects between injury and ROCK inhibition affected cell shape (area, oblate and prolate ellipticity, volume).

To understand how Y-27632 altered the response of muSCs to injury, cell shape and movement were compared between conditions (figure 3*i*; electronic supplementary material, figure S3A,B). In uninjured pax7a:egfp larvae, inhibition of ROCK caused increased sphericity and decreased cell displacement (delta displacement length), but had no other effect on shape or movement. In injured myotomes cells showed a reduction in cell volume and area as well as an increased oblate ellipticity and decreased prolate ellipticity, suggesting cells were less elongated. These shape changes in response to injury were altered in the presence of Y-27632: cell volume and area were increased compared to control animals, and prolate ellipticity was increased (electronic supplementary material, figure S9). These changes suggest inhibition of ROCK resulted in cells responding to injury adopting a more elongated shape. Addition of Y-27632 did not affect instantaneous speed in uninjured animals; in injured animals inhibition of ROCK resulted in an increased speed relative to untreated controls. Relative displacement was reduced by addition of Y-27632 in uninjured animals, but there was no affect in injured animals. However, there was a significant increase in relative cell displacement in injured animals compared to uninjured animals treated with Y-27632, suggesting that inhibition of ROCK results in increased movement of cells in the context of an injury.

To understand whether cell shape and movement show differences over time as a consequence of ROCK inhibition we used multiple regression models. Mixed-effect multiple linear regression models were tested that incorporated a term describing the interaction of time with the measure of shape or movement. Models that did not include a time interaction were used to assess measures of movement (delta displacement, displacement in *x*, *y* or *z*).

In uninjured muscle we found that application of Y-27632 (class 2) resulted in lower changes to surface area and cell volume over time (electronic supplementary material, table S3, figure S4). Rate of change for cell displacement was increased by ROCK inhibition, but rate of change to instantaneous speed was significantly reduced.

When comparing injured to uninjured muscle, volume showed no significant difference as a function of time, but surface area showed an increased rate of change in injured muscle (compare class 1 to class 3). By contrast, prolate ellipticity, oblate ellipticity and sphericity all showed lower rates of change in cells within injured myotomes compared to those in uninjured muscle. This reveals surface area fluctuated more over time as a consequence of the injury compared to uninjured controls, but overall cell shape showed less fluctuation over time. The rate of cell displacement showed an increased rate of change, whereas instantaneous speed showed a lower rate of change over time in injured compared to uninjured muscle. This suggests cells show a more variable movement in injured tissue but have a more constant movement relative to uninjured tissue.

The models reveal that Y-27632 treatment affected how cell shape and movement change over time in a similar manner in both injured and injured myotomes. In both conditions we found that Y-27632 treatment resulted in cells showing an increased rate of change to cell shape associated with an elongated morphology (decrease in oblate ellipticity, increase in prolate ellipticity, reduced sphericity). This indicates that inhibition of ROCK results in cells showing a more variable cell shape over time. The rate of change to instantaneous speed was reduced in the presence of Y-27632, suggesting cells adopted a more constant movement when RhoA signalling is inhibited (electronic supplementary material, figure S4).

### 3.4. Inhibition of ROCK alters adhesion dynamics in muSCs

**Figure 4.**
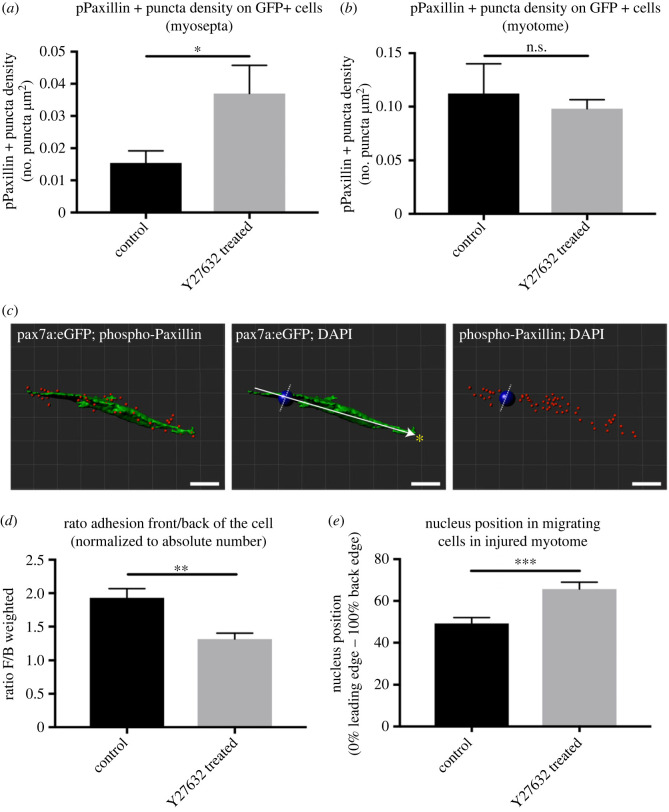
Measures of pPaxillin distribution in GFP+ cells of injured pax7a:egfp larvae in the presence or absence of 50 µM Y-27632. pPaxillin density within GFP+ cells at the myoseptum (*a*) and within the myotome (*b*) was measured by defining puncta per µm2 within a GFP+ cell. pPaxillin puncta distribution was measured along the longest axis of the GFP+ cell (defined as the axis towards the injury, arrow) relative to the nucleus (blue ball) in the front, *F*, or back, *B*, of the cell (*c*). A ratio was calculated from the number of puncta at the front and back normalized to the total number of puncta per cell for untreated animals or those treated with Y-27632 (*d*). The position of the nucleus was measured along the axis and expressed as a % relative to the leading (anterior) or trailing (posterior) edge. In animals treated with Y-27632 the relative position of the nucleus was more posteriorly located in GFP+ cells. Significance of difference was determined by an unpaired Student's *t*-test (***p < 0.001, **p < 0.01, *p < 0.05, N.S. *p* > 0.05) with *n* = 3 animals per condition. Scale bar 10 µm (*c*).

RhoA is important for stabilizing the formations of nascent adhesions allowing their maturation into focal adhesions by causing phosphorylation of FAK and Paxillin [64,65]. We hypothesised that inhibition of ROCK would therefore cause a loss of cell adhesions on muSCs resident at the myoseptum. To test this the distribution of focal adhesions within GFP+ cells of pax7a:egfp larvae was detected by immunolabelling with anti-pPaxillin on uninjured or injured larvae treated with Y-27632 or not treated (electronic supplementary material, figure S5). There was an increased density of pPaxillin + adhesions in muSCs resident at the myoseptum in Y-27632 treated larvae compared to untreated controls in contrast to our hypothesis (figure 4*a*). Y-27632 treatment also affected pPaxillin + puncta size and clustering in muSCs at the myoseptum suggesting that adhesions are more dispersed and smaller (electronic supplementary material, figure S6A,B).

We then evaluated whether inhibition of ROCK altered the distribution of focal adhesions in migrating muSCs within the myotome. There were no differences in the density of pPaxillin + puncta in migratory muSCs following Y-27632 treatment (figure 4*b*). However, clustering of adhesions was reduced, adhesions were smaller and more spherical than controls, suggesting an impaired formation of adhesions occurred as a consequence of ROCK inhibition (electronic supplementary material, figure S6C–G). As RhoA signalling has been shown to confer polarity to migratory cells we examined whether the distribution of pPaxillin + puncta was altered relative to the axis of migration in muSCs treated with Y-27632. The ratio of the number of puncta was calculated for the front (leading edge) and back (trailing edge) of the cell relative to the nucleus along the axis of cell migration towards the site of injury (figure 4*c*). There was a clear difference in the distribution of adhesions between muSCs responding to injury in control animals, with more focal adhesions at the leading edge of cells compared to those in animals treated with Y-27632 (figure 4*d*).

One consequence of ROCK inhibition in cells cultured in two dimension is a loss of cortical contractility and failure to move the nucleus [27]. We therefore examined nuclear positioning relative to the axis of migration of muSCs and found that the nucleus was more often located towards the trailing edge of the cell in Y-27632 treated animals than control animals (figure 4*e*). This reveals that nuclear displacement is reduced in migratory muSCs lacking ROCK activity.

A loss of RhoA signalling may lead to reduced mechanotransduction signalling via YAP/TAZ in the Hippo pathway, required for focal adhesion formation [66]. We therefore examined expression of zebrafish orthologues of three genes described as transcriptional targets of YAP/TAZ: *cgtf, cyr61* and *gli2a* [67]. We noted there was reduced expression of *cgtf* and *cyr61*, but not *gli2a*, in response to Y-27632 treatment (electronic supplementary material, figure S10).

### 3.5. ROCK inhibition impairs myogenesis

Factors affecting whether a cell will initiate proliferation or differentiation include the force transmitted to the nucleus as a consequence of cell adhesion or cell migratory behaviour. Having established that muSC cell polarity, adhesion and movement were affected by inhibition of ROCK we then investigated whether cell division or differentiation were also affected.

**Figure 5.**
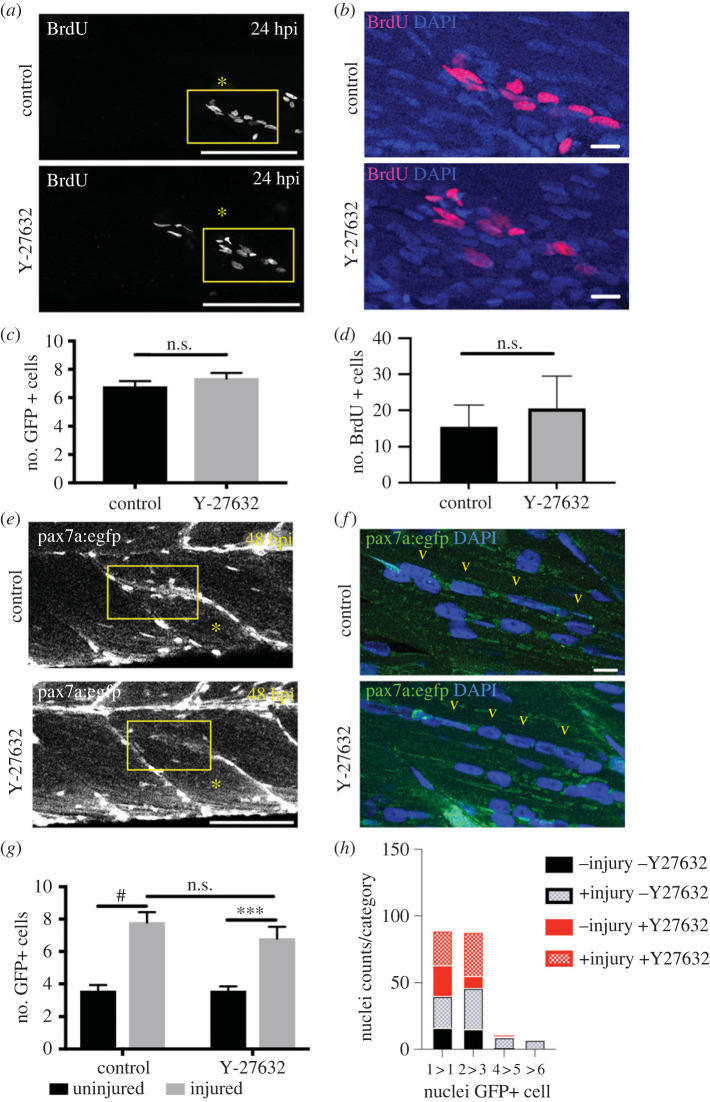
ROCK inhibition does not impair myogenesis during regeneration. Control injured pax7a:egfp larvae (injury location marked by asterisk) or those treated with 10 µM Y-27632 were exposed to BrdU for 24 h (*a*, enlarged area in *b*). Quantification of GFP+ cells (*c*) and BrdU + cells (*d*) in control and Y-27632 treated pax7a:egfp animals at 24 hpi. The number of GFP+ cells and number of nuclei per GFP+ cells was assessed at 48 hpi in control and Y-27632 treated animals (*e*, enlarged area in *f*). Comparison of the relative number of GFP+ cells in regenerating muscle of control animals compared to those treated with Y-27632 (*g*). Relative nuclear number per newly regenerating GFP + fibre is shown for control and Y-27632 treated animals and represented as classes with 1 nuclei (1 > 1), between 2 to 3 (2 > 3), between 4 to 5 (4 > 5) or more than 6 nuclei per GFP + myofibre (*h*). Significance was calculated using *t*-test with Bonferroni correction (*c,d*), by 2-way ANOVA with Tukey's *post-hoc* correction (*g*) or by a Chi-squared test (*h*) with *n* = 3 animals per condition. Scale bars 100 µm (*a,e*), 10 µm (*b,f*).

Injury results in an increased number of GFP + muSCs at 24 hpi in the pax7a:egfp line [16]. Y-27632 treatment did not result in a significantly different number of GFP + muSCs at 24 hpi (figure 5*c*). An examination of cell proliferation by BrdU labelling revealed no significant difference in the number of BrdU + cells in the myotome after injury between control and Y-27632 treated animals (figure 5*a,b,d*).

Rho, Rac and Cdc42 signalling drive a variety of myoblast behaviours including migration and fusion [68]. To determine how extended inhibition of ROCK affects the response of muSCs to injury we treated larvae with Y-27632 for 48 h after injury and assayed the distribution of GFP+ cells (figure 5*e,f*). We noted that there was no significant difference in the number of GFP+ cells at 48 hpi between controls and ROCK inhibited larvae (figure 5*g*). We therefore quantified the number of myonuclei per GFP+ cell in the myotome and tested for differences between the relative frequency of GFP + myofibres with 1, 2–3, 4–5 or greater than 6 nuclei using a Chi squared test (figure 5*h*). There was no significant difference between the relative frequency of myonuclei per GFP + myofibre between control and Y-27632 treated uninjured animals, but there was significantly fewer myonuclei per GFP + myofibre in injured larvae treated with Y-27632 compared to control injured animals (*p* < 0.01).

**Figure 6.**
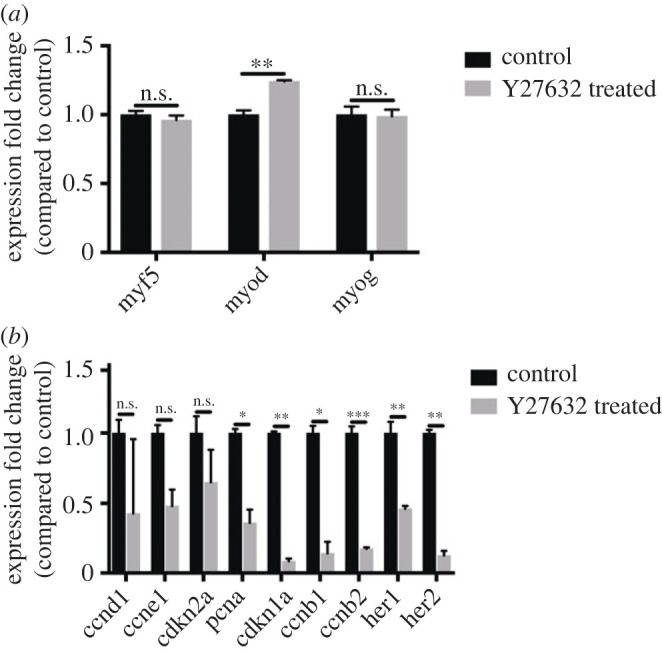
ROCK inhibition impairs myogenesis. Gene expression in regenerating myotomes of control and Y-27632 treated larvae was examined by qPCR at 24 hpi. Myogenic genes *myf5*, *myod* and *myogenin* were compared, but only *myod* showed an upregulation in Y-27632 treated tissue (*a*). Cell cycle associated genes *pcna*, *cdkn1a*, *ccnb1*, *ccnb2* and Notch activated genes *her1* and *her2* were downregulated in Y-27632 treated tissue, but *ccnd1*, *ccne1*, *cdkn2a* did not show significant differences (*b*). The difference in gene expression between control and Y-27632 treated tissue was tested by comparing ΔCt values using an unpaired *t*-test with Bonferroni correction with significance assumed where *p* < 0.05 (***p < 0.001, **p < 0.01, *p < 0.05, N.S. *p* > 0.05).

To understand how ROCK inhibition affects myogenesis during regeneration we evaluated gene expression in Y-27632 treated larvae at 24 hpi by *in situ* hybridization. Expression of *myf5*, *myod* and *myogenin* were all apparent at the site of injury with no clear differences between ROCK inhibitor treated compared to control animals (electronic supplementary material, figure S7A–F). Using quantitative reverse transcription polymerase chain reaction (qRT-PCR) we measured expression of myogenic genes in regenerating tissue at 24 hpi. We observed that *myod* expression was upregulated in response to ROCK inhibition but there was no change to *myf5* or *myog* expression (figure 6*a*). To determine whether this reflects a commitment to differentiation we examined genes implicated in cell cycle progression and proliferation. We observed that many genes involved in proliferation including *pcna*, *cyclinB1, cyclinB2* and *cdkn1a* were downregulated in Y-27632 treated larvae (figure 6*b*). By contrast *cyclin D1, cyclinE1* and *cdkn2a* were not affected. We also observed two Notch responsive genes *her1* and *her2* were downregulated in animals exposed to Y-27632.

**Figure 7.**
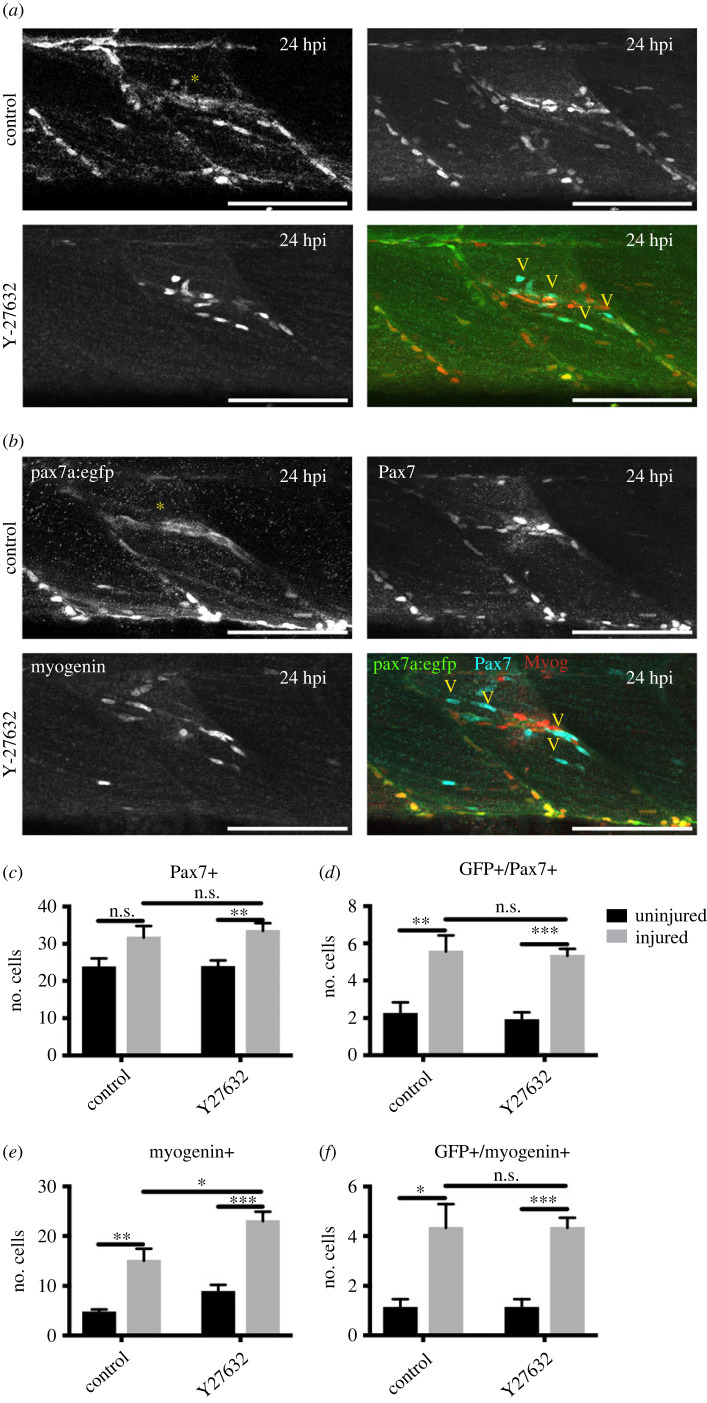
Myogenesis during regeneration of pax7a:egfp larvae was quantified in the absence (*a*) or presence (*b*) of Y-27632 by immunolabelling with antibodies to Pax7 (arrowheads), myogenin and GFP. Cells expressing Pax7+, myogenin, pax7a:egfp (GFP+) were counted in injured or uninjured myotomes at 24 hpi (*c–f*). Significance was tested by unpaired *t*-tests (*p < 0.05, **p < 0.01, ***p < 0.001) with *n* = 5 animals per condition. Scale bars 50 µm.

To determine whether muSC commitment to myogenesis is affected in response to ROCK inhibition we quantified the number of myoblasts expressing Pax7 or myogenin protein relative to expression of pax7a:egfp in the myotome of larvae at 24 hpi (figure 7*a,b*). In uninjured larvae there was no difference in the number of cells expressing GFP, Pax7 or myogenin between ROCK inhibitor treatments compared to controls (figure 7*c–f*). Likewise, there was no difference in the number of GFP + myoblasts or Pax7 + muSCs between control and Y-27632 treated larvae following injury (figure 7*c,d*). Although the number of GFP + myoblasts expressing myogenin did not change, there was an increased overall number of myogenin + myoblasts in the myotome of injured larvae treated with ROCK inhibitor (figure 7*e,f*). Therefore, inhibition of RhoA activity results in increased differentiation of muSCs and downregulation of genes promoting cell cycle progression in the context of injury.

## 4. Discussion

Our current understanding of how muSC migration is regulated is mostly based on analyses *in vitro*, despite a recognition that the cues they experience *in vivo* are fundamentally different. We have therefore investigated the dynamic response of muSCs to muscle injury *in vivo* using the zebrafish and characterized the importance of the RhoA kinase, ROCK, in this process. Our analyses of muSC behaviour reveal that cells detach from the myoseptum, have a polarized distribution of phospho-Paxillin and migrate directionally to injured myofibres. Inhibition of ROCK results in muSCs adopting a more constant and rapid movement, which correlates with them assuming an elongated shape and loss of muSC polarization. This coincides with increased differentiation of muSCs and a reduction in the expression of cell cycle genes in the regenerating myotome. We infer from these results that ROCK is required for normal migration of muSCs in response to injury. When RhoA signalling is inhibited, the adhesion based mechanism of cell migration is perturbed and muSCs become more prone to differentiate. We interpret these changes to arise from altered muSC-extracellular matrix interactions with consequential changes to mechanical cues encountered by these cells. As a consequence we suggest that changes to migration are predictive of altered outcomes of muSCs, with a higher adhesion and slower migration associated with increased proliferation and reduced adhesion and faster migration predictive of increased differentiation.

### 4.1. Rhoa regulates mesenchymal migration of muSCs

The mode by which muSCs migrate differs depending on the system employed (*in vitro, in vivo*), age of the animal they are derived from (foetal, adult, aged) and environment they encounter (extracellular matrix molecules, signalling molecules). Descriptions of musCs migrating *in vitro* on cultured murine myofibres suggest they adopt an amoeboid mechanism of migration utilizing membrane blebbing [28,69]. *Ex vivo* imaging of muSCs cultured in three dimensional matrices also argue for a blebbing based migratory mechanism [23]. However, neither of these *in vitro* culture approaches retained the extracellular matrix, which is known to be a critical regulator of muSC function, nor the inherent tension experienced by myofibres, which is likely to be an important regulator of their behaviour [70]. There are clear similarities in the descriptions of cell behaviour from *in vivo* imaging of mouse and zebrafish muSCs, suggesting that there is a conserved mode of movement [45]. Cells in both mouse and fish show an elongated morphology with protrusions extending and retracting as they migrate in a punctuated manner [16,17,22,29,30,71]. We find that muSCs in zebrafish express focal adhesion proteins pPaxillin and Vinculin and these are organized in a polarized manner relative to the orientation of cell migration. The presence of focal adhesions proteins suggests that muSCs make contacts to the extracellular matrix through integrin-mediated contacts and that this facilitates their migration in a mesenchymal manner.

RhoA signalling regulates several aspects of cell migration, principally by activating myosin II and by inhibiting the actions of the related small GTPase Rac [65,72–74]. Rho kinase (ROCK) phosphorylates myosin II and is a key regulator of actinomyosin dynamics. In mesenchymal cells it is required for cell cortex contractility, driving cell migration, as well as stabilizing cell protrusions [75]. Inhibition of ROCK function in fibroblasts results in cells assuming an elongated morphology and displaying a reduced number of adhesions [65,73,76]. In C2C12 myoblasts inhibition of ROCK-2 resulted in increased migration concurrent with smaller focal adhesions [77]. We observe that ROCK inhibition causes muSCs to adopt a more elongated shape with a less polarized distribution of adhesions. Fibroblasts migrating in 3D cell-derived matrix show an altered mode of migration following inhibition of ROCK activity, with lamellipodia as the principal protrusion as opposed to lobopodia [46]. Mouse macrophages lacking RhoA/ RhoB function show an elongated morphology indicative of a loss of Rho-activated trailing edge retraction [78]. Although we established that cells are more elongated in the presence of Y-27632, we could not distinguish whether protrusions formed by muSCs in zebrafish larvae were more similar to lobopodia or lamellipodia in response to ROCK inhibition. A key element of the lobopodial mode of migration is the role of the nucleus as a myosin II-dependent nuclear piston [27,79]. In muSCs exposed to ROCK inhibitor the nucleus was posteriorly displaced, suggesting the nuclear piston is disrupted and implying a switch from lobopodial migration to a protrusive based mechanism driven by actin polymerization at the leading edge. C2C12 myoblasts exposed to Y-27632 show a similar switch to a protrusive mode of migration towards chemotactic cues concurrent with similar shape changes we identify in zebrafish [77]. This strongly argues that the default action of RhoA signalling is to promote a lobopodial mode of migration by muSCs and thereby control cell speed. We find that similar to treatment of C2C12 myoblasts with Y-27632, muSCs show an increased speed of migration with reduced variability in speed over time. We could not demonstrate that cells arrived at the injury site earlier as a consequence of ROCK inhibition, but this may be indicative of heterogeneity in behaviour between cells, or a delay in their active migration. Recent descriptions of muSCs examined in injured mouse muscle reveal similar changes to cell shape as we have described in zebrafish in response to ROCK inhibition. These include a more elongated cell morphology and changes to the cytoskeleton indicative of a disruption to the actinomyosin complex driving directional movement [32]. Our interpretation of these results are that muSCs use adhesion-mediated contacts to drive a punctate migratory behaviour. In an absence of RhoA signalling adhesion of muSCs to the extracellular matrix is reduced and cells show a more motile behaviour utilizing actin protrusions.

### 4.2. Attenuation of ROCK activity inhibits cell adhesion and force induced signalling within muSCs

Adhesion of mesenchymal cells is mediated via tethering of the actin cytoskeleton through integrins, that make contacts with proteins in the extracellular matrix such as fibronectin and laminins [55]. Transient adhesions are formed by integrin clustering and activation by Kindlin, Talin and Vinculin [80,81]. Contacts between clustered integrins and extracellular proteins such as fibronectin results in recruitment of further adhesion proteins including Paxillin and FAK. Focal adhesion (FA) maturation is RhoA-dependent and subsequent attachment to the actin cytoskeleton results in stabilization of the FA [82]. We observed puncta of Vinculin and pPaxillin in muSCs that were distributed in a polarized manner relative to the direction of cell migration. In the presence of ROCK inhibition pPaxillin + puncta were observed in muSCs suggesting FA formation was still occurring. The degree of adhesion is directly related to the amount of FA formed and there is a balance between the role of adhesions in enabling cell migration and causing cells to adhere too strongly to the substrate and inhibiting migration [83] Porcine muSCs plated on fibronectin migrated in a manner correlated with the relative expression of FAK and pPaxillin [84]. We could not detect a difference in the number of pPaxillin puncta in muSCs exposed to ROCK inhibitor, but did note the puncta were generally smaller, similar to reports from treatments of a variety of cell types treated with Y-27632 [85]. Although the number of pPaxillin + adhesions in muSCs was not different between control and ROCK inhibitor treated larvae, we did observe that their distribution relative to the axis of migration was altered. Specifically, adhesions were more evenly distributed across the cell surface in an absence of ROCK activity, in contrast to the enrichment of adhesions at the protrusive leading edge of the cell seen in control animals. Adhesion formation at the leading edge of the cell and disassembly at the trailing edge is a major mechanism for mesenchymal cell migration. Coupled with the aberrant positioning of the nucleus, these phenotypes suggest that RhoA activity is required for promoting adhesion formation and cortical contraction thereby driving muSC migration. The more elongated shape of the cells coupled with the loss of adhesion polarization suggests that muSCs are less adherent in an absence of RhoA signalling. This is correlated with an increased instantaneous speed of muSCs when responding to injury when ROCK activity is inhibited. *In vitro*, a loss of ROCK activity in fibroblasts or C2C12 myoblasts causes cells to adopt a gliding movement, concomitant with reductions of focal adhesion size and reduced cortical contractility [27,77]. Our results agree with these *in vitro* observations, indicating that RhoA acts to promote cortical contractility and stabilize adhesion formation, inducing a punctate, adhesion-dependent migratory behaviour.

### 4.3. Cell migration and proliferation are coupled processes in muSCs

A link between cell migration and proliferation has been described for a number of cell types [86,87]. The stiffness of the substrate that mammalian muSCs adhere to has been shown to play an important role in dictating their rates of proliferation or differentiation [38]. Stiffer substrates promote differentiation, whereas more pliant substrates promote proliferation. However, a study of muSCs responses to damaged myofibres revealed that although these display an increased stiffness, there is also more proliferation [39]. The balance between substrate stiffness and tension induced in the cell is therefore likely to dictate how muSCs respond to tissue injury. We note that inhibition of ROCK results in downregulation of cell cycle gene expression in the regenerating muscle. We were not able to demonstrate a difference to the number of BrdU incorporating cells in Y-27632 treated animals, suggesting that S-phase in muSCs was not affected. A closer examination of cell cycle genes affected by RhoA inhibition reveals both *cyclin B1*, *cyclin B2* and *cdkn1a* are downregulated. These are associated with regulation of G2/ M in myoblasts [88] and Cdk1 is required for muSC proliferation [89]. By contrast *cyclin D1* and *cyclin E1*, associated with G1/S phase did not show any difference in expression. Downregulation of G2/M associated genes coincided with elevated numbers of myoblasts expressing Myogenin, a marker of differentiation. Several studies have demonstrated that Cdkn1 and Cdkn2 phosphorylate Myod leading to repression of Myod target genes at the G2/M phase of the cell cycle [90,91]. Our observation that gene expression associated with G2/M progression is reduced, coupled with increased expression of Myogenin, a Myod transcriptional target, suggests RhoA inhibition results in muSCs preferentially exiting the cell cycle at G2/M and differentiating as a consequence of increased Myod activity.

**Figure 8.**
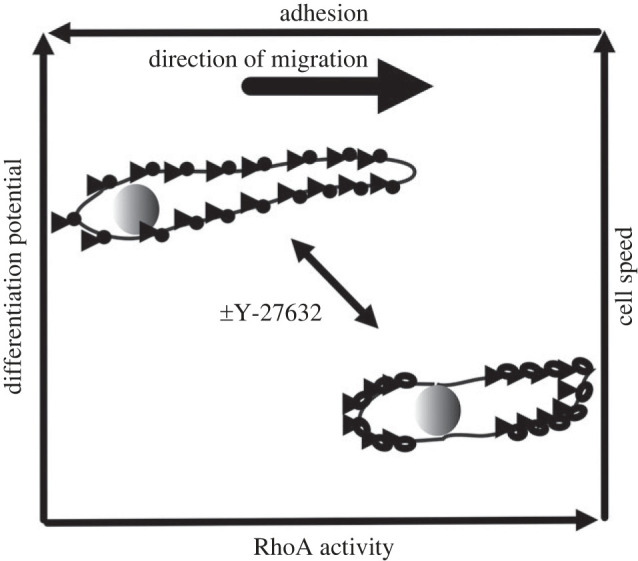
A model for RhoA-dependent migration of muSCs. Migratory muSCs show a RhoA-dependent cell cortex contractility that drives nuclear positioning and cell migration. In an absence of ROCK activity caused by Y-27632, focal adhesions are smaller, less clustered and less polarized along the axis of cell migration leading to reduced adhesion to the extracellular matrix. This correlates with an increased differentiation potential and more rapid migratory behaviour of muSCs.

Inhibition of ROCK causes diminished myosin-actin contractility of the cell and hence reduced cortical tension [72] The adhesions in muSCs lacking ROCK activity were smaller and less polarized, suggesting an overall reduction in tension forces experienced by the migrating cell. By manipulating substrate mediated tension and cytoskeletal function Mih *et al.* found that proliferation of mesenchymal cells could be adjusted, highlighting a mechanism for coupling tension and cell proliferation [85]. Inhibition of ROCK signalling in endothelial cells lacking YAP/TAZ activity resulted in increased motility and smaller focal adhesions [92]. The authors argued this phenotype was a consequence of reduced tension in the cells, enabling a more rapid turnover of adhesions and hence faster movement. We likewise observe smaller adhesions, increased motility and downregulation of genes regulating cell cycle progression in zebrafish with inhibited ROCK activity. The relationship between mechanotransduction and cell migration in muSCs can potentially be explained in a model in which tension is propagated through the cytoskeleton, regulating FA turnover, and conveying force-activated changes to gene expression from the local environment. Our results suggest that ROCK-dependent cortical tension is required for muSC proliferation and to prevent differentiation *in vivo* (figure 8). We find support for this model from the many *in vitro* studies showing that increased tension experienced by muSCs promotes proliferation, whereas reduced tension is associated with differentiation [38,39,93,94].

Given the role of Yap/Taz signalling in mediating cell tension and regulating cell proliferation it is possible that the phenotypes we observe in muSCs lacking RhoA activity are a consequence of reduced Yap signalling. Inhibition of Yap/Taz activity is associated with lower cell proliferation and loss of polarized focal adhesion distribution in migrating endothelial cells [92]. Our results show that inhibition of RhoA results in similar changes to muSCs. Furthermore we note that expression of YAP/TAZ regulated genes *ctgf* and *cyr61* are downregulated in muscle of larvae treated with Y-27632. This suggests RhoA activity is required for promoting intracellular tension and maintaining polarized focal adhesions in migrating muSCs. Mechanotension then induces Yap activity, which promotes cell proliferation through upregulation of cell cycle genes, preventing a premature differentiation of muSCs as they migrate to the injury site. Potentially the phenotypes we observe in muSCs are a consequence of loss of ROCK activity in other cells. In adult muscle it has been shown that RhoA signalling is important for recruitment and fusion of muSCs to myofibres in response to increased load, via modulation of matrix remodelling enzymes [95]. Conditional loss of RhoA signalling in myofibres resulted in reduced muSC proliferation and differentiation in response to muscle loading. This appears to differ from our results which show changes to cell cycle gene expression, but no overall reduction in proliferation, and an increased tendency to differentiate. It is difficult to extrapolate findings from manipulating RhoA during load induced muscle growth to tissue injury as these involve different types of cell responses, but it would be intriguing to understand the importance of force-inducing signals from myofibres on muSCs. As we have shown previously by *ex vivo* imaging of muscle explants from mice, muSCs respond to myofibre stretch [96]. It is therefore likely that changes to RhoA-mediated signalling in myofibres would also affect resident muSCs.

### 4.4. Summary

Cell migration is a critical property enabling stem cells to respond to damage signals and repair tissue. During this process they encounter diverse cues from their environment that will affect their fate and ability to repair tissue, including mechanical signals mediated via the cytoskeleton. We show that muSCs responding to injury *in vivo* are affected by alterations to RhoA activity, a critical regulator of the cytoskeleton. Inhibition of RhoA results in muSCs adopting a more elongated shape, a reduced polarity and smaller focal adhesions. MuSCs also show a decreased expression of cell cycle genes associated with G2/M phase and are more prone to undergo differentiation. Our interpretation of these changes to muSC behaviour, shape and fate is that reduced mechanical signalling from the environment to muSCs results in a shift from proliferation to differentiation. This has implications for how muSC respond to tissue injury when mechanical forces from the environment are altered, with increased stiffness potentially inducing proliferation and reduced stiffness inducing differentiation. However, we have not demonstrated that this is due to autonomous changes to mechanosensing in muSCs. An alternative hypothesis is that changes to tension in the environment due to a reduction in myosin phosophorylation within myofibres as a consequence of ROCK inhibition induces altered mechanical signalling to muSCs. Cell specific manipulation of cytoskeletal proteins would resolve whether ROCK activity within muSCs is important for regulating their proliferation or differentiation.

In contexts such as ageing and muscle dystrophies, the stiffness of the ECM is increased and muSCs show a reduced regenerative potential [97]. *In vitro* observations of muSCs on isolated myofibres from ageing mice reveal that cell migration is reduced and membrane retraction is impaired [69]. These features are reminiscent of those we and others have described in progenitor cells with reduced RhoA activity. It is intriguing to consider whether aspects of muSC behaviour in ageing muscle might be rejuvenated by enhancing migration through modulation of RhoA signalling.

## 5. Conclusion

Our results reveal for the first time that muSC migration and cell cycle progression are regulated by interactions with the local environment during regeneration *in vivo*. We demonstrate that changes to cytoskeletal-mediated forces can skew cells fates towards proliferation or differentiation and that this is linked to cell behaviour.

## Data Availability

Projections of time-lapsed imaging datasets and Imaris processed files are provided in electronic supplementary material [98].
